# Community conservatism is widespread across microbial phyla and environments

**DOI:** 10.1038/s41559-025-02957-4

**Published:** 2026-01-16

**Authors:** Lukas Malfertheiner, Janko Tackmann, João Frederico Matias Rodrigues, Christian von Mering

**Affiliations:** Department of Molecular Life Sciences and Swiss Institute of Bioinformatics, University of Zurich, Zurich, Switzerland

**Keywords:** Computational biology and bioinformatics, Speciation, Microbial ecology, Microbial communities

## Abstract

Phylogenetic signal describes the tendency of related organisms to resemble each other in morphology and function. Related organisms tend to also live in similar ecological niches, which is termed niche conservatism. The concepts of both phylogenetic signal and niche conservatism are widely used to understand crucial aspects of evolution and speciation, and they are well established in animals and plants. However, although assumed to be present, the extension of these concepts to microorganisms is challenging to assess. Here we hypothesize that two closely related microbial species should be found in samples with similar community compositions, reflecting their ecological similarity. We propose ‘community conservatism’ to refer to this phenomenon and leverage a database with millions of samples and hundreds of thousands of pairs of microorganisms to assess their relatedness and the similarity of the communities they occupy. Our findings reveal that community conservatism can be observed globally in all environments and phyla tested, over nearly all taxonomic ranks, but to varying extents. Analysing community conservatism shows promise to advance our understanding of evolution, speciation and the mechanisms governing community assembly in microorganisms. Furthermore, we propose that it can be used to reintegrate ecological parameters into operational taxonomic unit delimitation.

## Abstract (web summary)

This study reveals that closely related microorganisms tend to inhabit similar communities across all major environments and phyla. The authors term this phenomenon ‘community conservatism’, extending the ecological concepts of phylogenetic signal and niche conservatism to the microbial world.

## Main

**Fig. 1:**
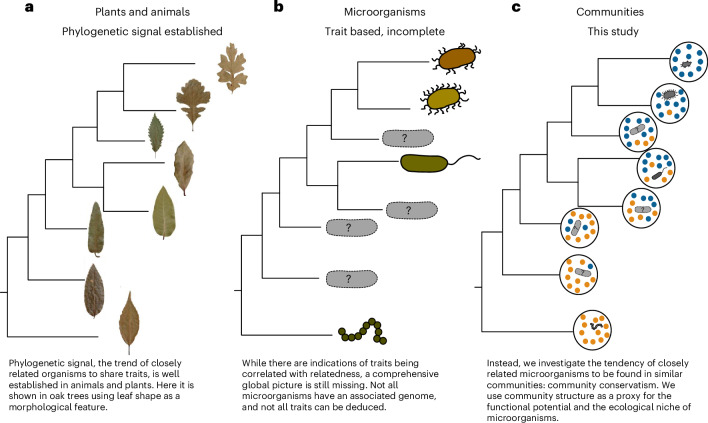
Community composition to measure evolutionary patterns in microorganisms. **a**, The leaf shape of oak trees is a morphological feature that shows a strong phylogenetic signal. Closely related species have similar leaf shapes, whereas more distantly related species have larger differences. **b**, In bacteria, there are also indications that traits are phylogenetically conserved, as in ref. ^28^. However, we often do not know enough about the morphology or physiology of these organisms, as most of them remain uncultured. **c**, We propose community conservatism as an alternative approach: instead of comparing bacterial species directly in terms of physiology or morphology, we assume that if they are related (and thus potentially have a similar function and occupy a similar ecological niche), then their community composition will also be similar. Images in **a** adapted with permission from ref. ^6^, PNAS.

Organisms tend to retain their ancestral ecological niches over time^1,2^. This so-called niche conservatism is often discussed in the context of a broader concept, phylogenetic signal, in which closely related species tend to resemble each other morphologically and functionally^3^. Numerous studies have shown niche conservatism and phylogenetic signal in animals and plants^4–6^. Therein, the analysis and distribution of various traits, such as habitat preferences, morphology (for example, leaf shape; Fig. 1a) and physiology, shed light on crucial aspects of evolution, including speciation. In addition, these studies help to predict how eukaryotes may adapt to rising challenges such as the spread of invasive species or climate change^7,8^.

Apart from animals and plants, microorganisms also fulfil crucial roles in almost all areas of life, from driving biogeochemical cycles to influencing human health and diseases^9–11^. Despite their importance, much less is known about the ecology and long-term evolution of microorganisms: even the concept of species in microorganisms itself is a long-standing matter of debate^12–14^. In addition, their phenotypes and habitats are more difficult to assess, compared with animals and plants, especially considering that many cannot yet be cultivated under controlled conditions^15^. Regardless of these difficulties, the assumption that phylogenetic signal and niche conservatism are present globally in microorganisms is used in many popular algorithms, such as UniFrac and Phylogenetic Interaction-Adjusted index (PINA)^16,17^. Characterizing microbial niche conservatism and phylogenetic signal on a global scale is thus crucial, yet challenging owing to the lack of information about the characteristics of uncultured microorganisms^10,18^. While related microorganisms have been predicted to more frequently interact with one another (phylogenetic assortativity)^19–21^ and at least a broad social community preference is detectable in microorganisms^22^, only limited direct evidence exists for niche conservatism and phylogenetic signal^23–26^, which is generally restricted to selected environments or taxa. For instance, studies indicate that some genome-derived traits can be conserved over long time periods in microorganisms (Fig. 1b)^27,28^.

Here we look for an alternative to trait-based assessments of ecology, as environmental parameters are often not known and morphological features are scarce. We focus on the high-quality data that we have: millions of DNA-sequenced microbial community samples from all over the globe, and phylogenetic marker genes such as 16S rRNA that enable us to estimate in which communities a given microbial species occurs. Community structure can accurately distinguish different ecological niches^29–33^ and has successfully been used to determine niche ranges in generalist and specialist animals^34^ and microorganisms^22^.

Following this line of work, we here treat community composition as a proxy for the realized niche of a microorganism—the latter being determined through multiple, often unknown effects, ranging from the abiotic environment to microbial interactions. Thus, we hypothesized that by using a community-centric approach, we can approximate phylogenetic signal and niche conservatism in microorganisms by analysing the tendency of closely related organisms to occur in similar communities (Fig. 1c).

We show with an extensive analysis that more closely related taxa indeed occur in more similar communities. Remarkably, this trend is consistently detectable in all investigated phyla and environments. We suggest the term ‘community conservatism’ for this phenomenon and show that remnants of microbial community preferences can be traced back billions of years. Furthermore, we show varying trends of community conservatism in different phyla, infer generalism- and specialism-specific signals, and provide hundreds of operational taxonomic unit (OTU) pairs with potential interest for diverse research areas. Lastly, we outline the potential use of community conservatism as a second parameter—next to sequence similarity—in OTU clustering, to reintegrate ecological information in the future.

## Results and discussion

### Community structure as a proxy for niches and functional potential

We investigated global microbiomes using the MicrobeAtlas^35^ project (https://www.microbeatlas.org), an online database from which we used a filtered set of 1,153,349 environmental microbiome sequencing samples. MicrobeAtlas clusters microbial taxa into hierarchical OTUs using different similarity thresholds (from 90% to 99% full-length 16S rRNA sequence similarity, whereas 97–99% traditionally correspond to ‘species-level’ taxonomic groups^36^). By using such standardized occurrence data on a global scale, classical ecological questions can be investigated, such as the function of the ocean microbiome or which microorganisms are crucial for dissolving organic carbon^31,37,38^.

**Fig. 2:**
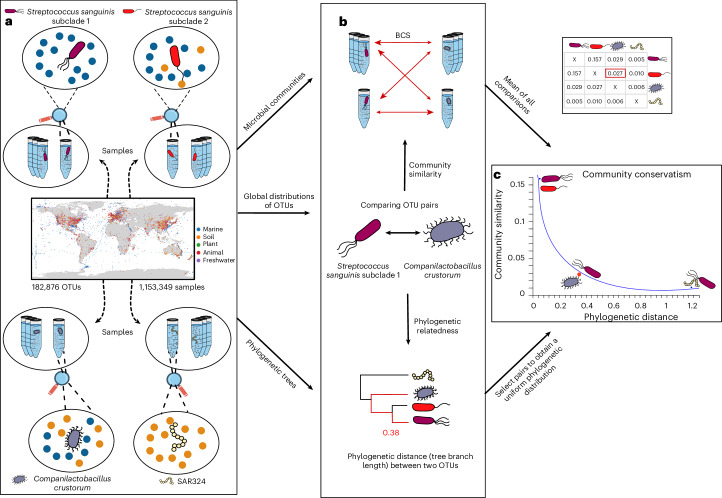
Analysis workflow. **a**, Illustration of the workflow using four selected example OTUs: two closely related *S. sanguinis* subclades, *C. crustorus* still belonging to the same phylum (Bacillota) but a different family and an only distantly related SAR324 strain. Within the MicrobeAtlas database, all microbial sequencing samples (and their communities, respectively) matching strict quality filters are retained for testing, resulting in a global picture of the communities in which each OTU occurs (1,153,349 samples, 182,876 99% OTUs). **b**, We compared OTU pairs using two main parameters: their relatedness, estimated by the tree branch length from a 16S rRNA tree, and the average of all beta diversity calculations (Bray-Curtis similarity, BCS) from the communities in which they are found. **c**, After selecting test pairs following a uniform phylogenetic distribution, we visualize three selected pairs in a scatter plot. Each dot is one OTU–OTU pair, with their relatedness shown on the *x* axis and the average similarity of their communities on the *y* axis. Pairs that are closely related and show a large community conservatism are expected on the top left, and distantly related pairs with different communities, on the bottom right. Source data

We worked with 182,876 OTUs defined at 99% sequence similarity (16S rRNA similarity) and initially assessed in which samples these OTUs occur globally (Fig. 2a). Next, we compared OTUs in a pairwise manner and calculated two main parameters for each pair: (1) the relatedness of the involved OTUs and (2) the similarity of the communities in which they occur (Fig. 2b). Relatedness is estimated from a large phylogenetic tree, from which we randomly sampled pairs of OTUs to obtain a uniform distribution of phylogenetic distances (Supplementary Fig. 1). We then assessed the beta diversity of all communities in which we detected them. For each pair, all samples containing the first OTU are compared with all samples containing the second OTU, measured as average pairwise Bray–Curtis similarity (BCS: 1 − Bray–Curtis dissimilarity). Previous work showed that BCS can adequately distinguish ecological niches^22^ and it can be efficiently computed at scale using optimized software^39^. Lastly, pairwise plotting of relatedness and average community similarity values of each OTU pair—combined with curve fitting—is used to assess the community conservatism signal (Fig. 2c). Our pairwise approach more explicitly assesses the relatedness of microorganisms independent of taxonomic binning and allows us—separately for each lineage—to quantify how community structure changes over evolutionary timescales, extending earlier work^22^.

To illustrate the general workflow, we compared four example OTUs with one another, at varying levels of relatedness: *Streptococcus sanguinis* subclade 1, *S. sanguinis* subclade 2, *Companilactobacillus crustorus* and Sar324 (Fig. 2). The two *S. sanguinis* clades, belonging to the Bacillota, are closely related commensals found in the oral cavity of humans^40^ with similar communities (average BCS of 0.15). *C. crustorus* is a more distantly related Bacillota OTU found in diverse environments, including the human microbiome^41,42^. Thus, despite sharing some community members (BCS 0.03), *C. crustorus* occupies different niches and appears to be more of a generalist. Lastly, SAR324 is a predominately marine bacterium that is found in different layers of the ocean^43^. It is only distantly related to the other OTUs, and as expected also its inhabited communities are very dissimilar (BCS 0.005). Hence, our hypothesis that more closely related OTU pairs occur in similar communities is supported in this small example.

### Community conservatism is present on a global scale

**Extended Data Fig. 1:**
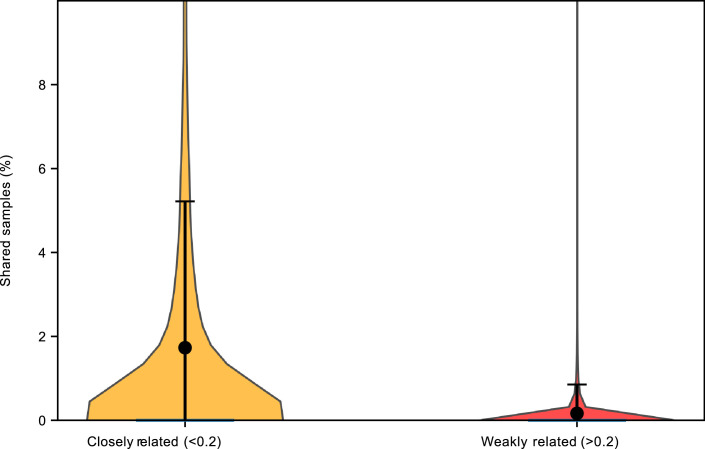
Closely related OTUs tend to co-occur across samples. The percentage of shared samples in closely related OTU-pairs (phylogenetic tree branch length <= 0.2, n = 2,716) is shown on the left-hand side in yellow. Weakly related OTU-pairs (phylogenetic tree branch length > 0.2, n = 22,284) are on the right side in red. The black dot denotes the mean percentage of shared samples ± standard deviation shown as black lines. Source data

To extend this workflow to a global scale, we chose 25,000 strictly quality-filtered, taxonomically annotated 99% OTU pairs. We first assessed their sample-by-sample co-occurrence, showing that related OTUs tend to occur more frequently in the same samples (Extended Data Fig. 1). While probably biologically relevant, this signal would compound our observations by inflating beta diversity values when comparing identical samples. To mitigate this effect, we chose a conservative approach and compared only samples that were not identical and did not belong to the same research project (that is, do not share the same ‘project ID’ at the Sequence Read Archive).

**Fig. 3:**
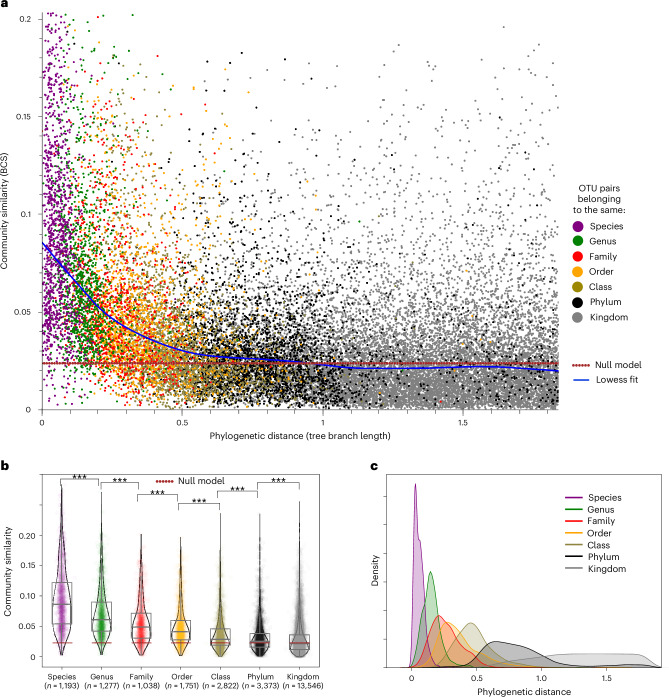
Community conservatism is present globally in microorganisms. **a**, Community similarity tends to fall as phylogenetic distance increases, visualized here through 25,000 OTU pairs with available taxonomic annotation to the species level; locally weighted scatter plot smoothing (lowess) fit and random expectation are shown as blue and red dotted lines, respectively. Each dot corresponds to one OTU pair coloured according to their most specific shared taxonomic rank, with their relatedness (tree branch length) shown on the *x* axis and the average similarity of their communities (Bray-Curtis similarity, BCS) on the *y* axis. **b**, All OTU pairs are binned based on the most specific taxonomic rank they share. Each dot corresponds to a pair, and the number of pairs per taxonomic bin corresponds to *n*. There are significant decreases in the community similarity between all taxonomic levels, down to the phylum level (***two-sided *P*_Mann–Whitney *U*_ < 1.0 × 10^−8^). Exact values are presented in Source Data. Each violin outlines the kernel density distribution of the data. Grey boxes indicate the interquartile range (IQR) and the median (horizontal line), while whiskers extend to 1.5 × IQR. **c**, Density plot showing the phylogenetic distance distribution of pairs belonging to the same taxonomic groups. Source data

**Extended Data Fig. 2:**
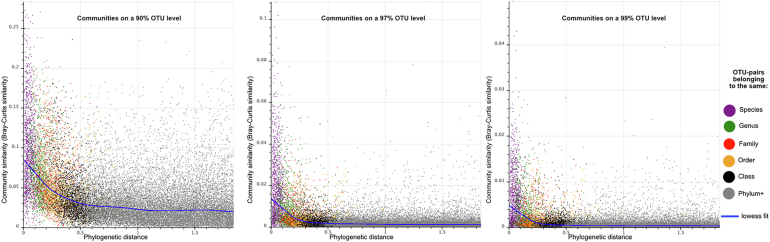
Different OTU-thresholds for the calculation of community similarities. Different granularity OTUs (90%, 97% and 99%) used to compute the beta diversity in the microbial communities (y-axis). Each dot corresponds to one OTU-pair colored according to their most specific shared taxonomic rank. Source data

We aimed to select the OTU threshold that best reflects the ecological niche for the computation of beta diversities. While we observed the same community conservatism trends with 90%, 97% or 99% OTU definitions (Extended Data Fig. 2), it has been hypothesized that microbial ecological niches are most clearly reflected at the species^44,45^ or genus level^9,46^. In our dataset, 90% sequence similarity between OTUs roughly corresponded to a genus- or family-level divergence^47^ (Supplementary Fig. 2). More sequence reads can be unambiguously assigned when using 90% OTUs; thus, we decided to use this level for all community similarity calculations (*y* axis in Fig. 3a) going forwards.

**Extended Data Fig. 3:**
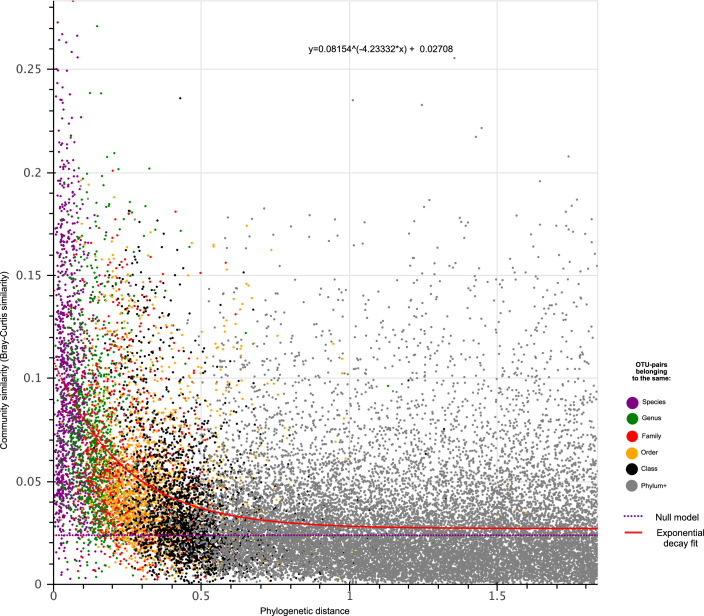
Community similarity follows an exponential decay with phylogenetic distance. Community similarity falls as phylogenetic distance increases, visualized here through 25,000 OUT pairs with available taxonomic annotation to species level: exponential fit (and formula) as well as random expectation are shown as red and purple dotted lines, respectively. Each dot corresponds to one OUT-pair colored according to their most specific shared taxonomic rank, with their relatedness shown on the x-axis and the average similarity of their communities (Bray-Curtis similarity) on the y-axis.

**Extended Data Fig. 4:**
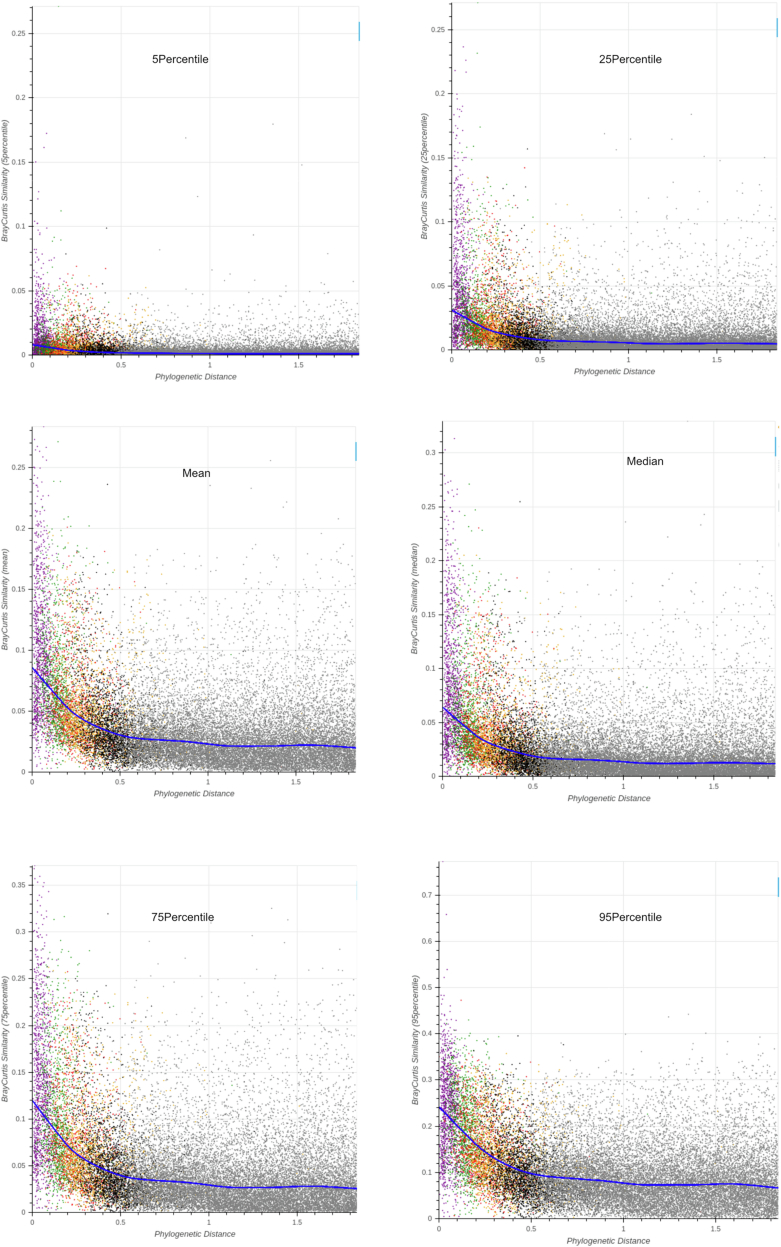
Community conservatism signal remains consistent when using different community similarity percentiles. Different percentiles of community similarities are shown here, in addition to the respective lowess fit as blue lines. Each dot corresponds to one OTU-pair colored according to their most specific shared taxonomic rank, with their relatedness shown on the x-axis and the average similarity of their communities (Bray-Curtis similarity) on the y-axis. Source data

**Extended Data Fig. 5:**
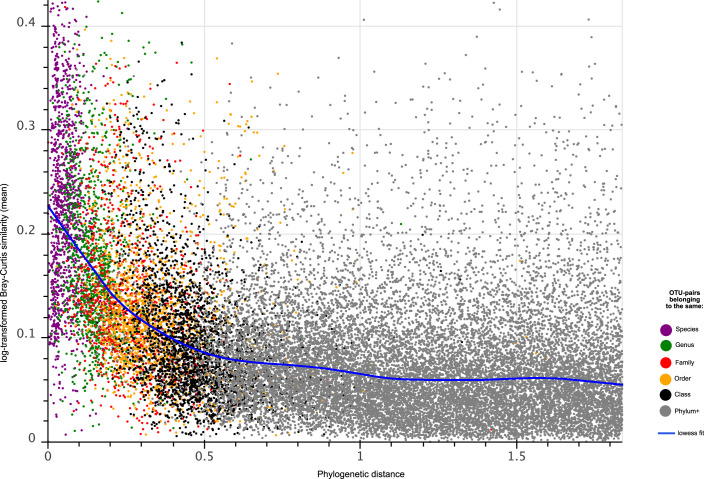
Community conservatism remains consistent when giving less emphasis to highly abundant OTUs. Community similarity falls as phylogenetic distance increases, visualized here through 25,000 OUT pairs with available taxonomic annotation to species level; lowess fit is shown as blue line. Each dot corresponds to one OUT-pair colored according to their most specific shared taxonomic rank, with their relatedness shown on the x-axis and the average similarity of their communities (log transformed Bray-Curtis similarity to mitigate any bias towards the most abundant OTUs) on the y-axis. Source data

Our results show the presence of community conservatism in microorganisms (Fig. 3a): OTU pairs that are more closely related (*x* axis, towards the left) are more similar in their communities (*y* axis, towards the top). To visualize this observation, we fitted a locally weighted scatter plot smoothing (lowess; Fig. 3a) as well as an exponential decay function (Extended Data Fig. 3) to the data. We found that both fitted curves strongly deviate from a null model based on expected average community similarities between random samples (exponential decay coefficient: −4.23). Trends remain similar when using medians or other percentiles to aggregate community similarities (Extended Data Fig. 4), or when log transforming the data before calculating beta diversity to exclude the possibility that the observed trend is mostly driven by highly abundant OTUs (Extended Data Fig. 5).

To obtain a statistical estimation of community conservatism, OTU pairs were binned according to their latest shared taxonomy. Significant deviation above the baseline was observed for each taxonomic level to the next, all the way up to the phylum level (*P*_Mann–Whitney *U*_ = 2.7 × 10^−23^; Fig. 3b). This indicates that community preference can be traced back to—and has potentially been transmitted across—billions of years^48,49^. The largest differences in the average BCS exist between the species and genus levels (*P*_Mann–Whitney *U*_ = 3.4 × 10^−37^), suggesting that species-level adaptations are particularly important for community preferences.

To test whether the assigned taxonomy is indeed reflected by phylogenetic distance, we checked how well the taxonomic relatedness of OTUs, based on available National Center for Biotechnology Information (NCBI) annotations, overlapped with the tree branch lengths that we use to estimate relatedness. Overall, taxonomic ranks follow phylogenetic distances as expected (Fig. 3c). However, it is also apparent that in some cases taxonomic classifications and 16S rRNA sequence similarities do not fully agree. This is consistent with known deviations between trait-based taxonomies and purely sequence-based clustering^50^.

**Extended Data Fig. 6:**
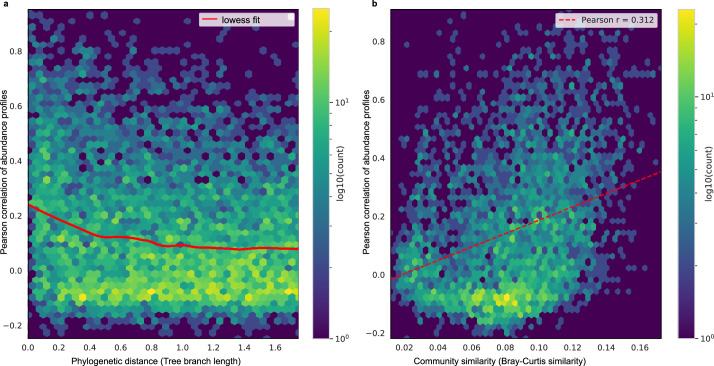
Community conservatism is recurrent in longitudinal sampling. a. Closely related OTU-pairs (x-axis, left) also show a higher correlation of their abundance profiles (y-axis, higher=more similar) over the course of multiple years within the Hawaii Ocean Time-series (HOT). The red line shows a lowess fit to the data. b. These marine OTUs with a higher correlation also tend to overall occur in more similar communities across the whole dataset (x-axis, right), with a Pearson correlation of 0.31 (p _pearson_ = 7.7e-96) shown with a red dotted line. Source data

We showed that OTU pairs belonging to the same species are often found in very similar communities, and hence, competition due to their overlapping niches might be expected. The coexistence of many direct competitors should not be feasible according to classical ecological models^51–53^ and can lead to phylogenetic overdispersion^54^. While this conflicts with our observation that closely related strains are also often co-occurring (Extended Data Fig. 1), there have been more observations showing the said co-occurrence^55–57^. Recent research has shown that horizontal gene transfer might alleviate the competition between related microbial species and allow the coexistence of many closely related competitors^58^. However, competition or exclusion of closely related microorganisms at strain-level resolution—which remains mostly undetectable in our 16S rRNA-based analysis—cannot be excluded. In support of our results, we checked whether community conservatism trends are recurrent within localized time-series data, spanning multiple years. To achieve this, we analysed samples belonging to the Hawaiian Ocean Time (HOT) series and analysed which OTUs show the highest correlations of their abundance profiles, indicating that they fluctuate together in different seasons and years. The OTU pairs with the highest Pearson correlation values also turn out to be more closely related (Extended Data Fig. 6a). Moreover, marine OTU pairs having correlated abundance profiles in the time series also occur in more similar communities outside the context of time-series experiments, in the global database within MicrobeAtlas (Extended Data Fig. 6b; *r* = 0.31, *P*_Pearson_ = 7.7 × 10^−96^).

### Environmental preferences are entangled with community conservatism

**Fig. 4:**
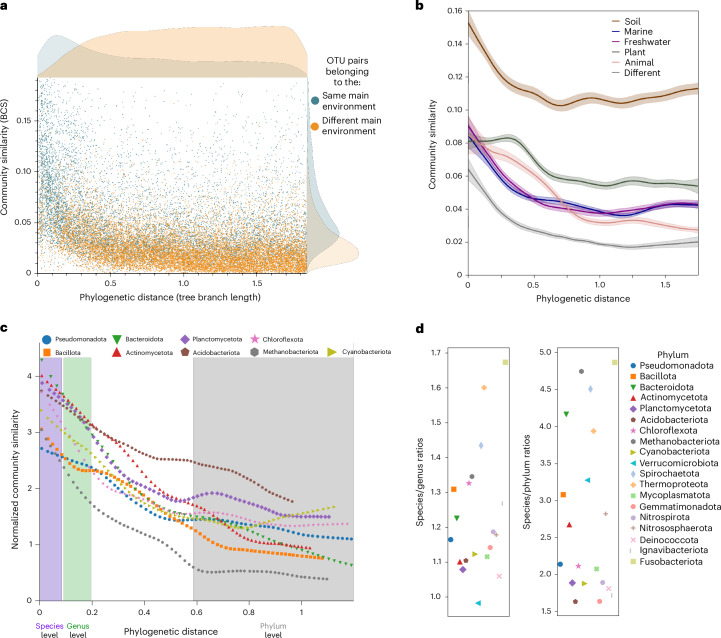
Environmental effects and phylum-level differences. **a**, The phylogenetic distance of 25,000 OTU pairs is plotted against the similarity of the communities they occupy. The pairs are coloured according to whether they share the same main annotated environment (blue) or are assigned to differing environments (orange). Bray-Curtis, BCS. **b**, Only OTU pairs belonging to the same given main environment (soil, marine, freshwater, soil or plant) or to different environments (grey) are compared, 25,000 pairs each. The solid lines represent the mean of 30 bootstrapped lowess fits. The shaded areas denote ±1.96 × standard deviation (approximate 95% confidence interval). **c**, All phyla with >3,000 available OTUs are shown here, and a lowess fit is created for each, calculated from 10,000 OTU pairs per phylum. The signal is normalized by environmental preference (Methods). In addition, taxonomic ranges (estimated from Fig. 3c) are indicated by colour shade (purple = species, green = genus, grey = phylum). **d**, Community conservatism ratios (curve steepness) calculated from the taxonomic bins (**c**) of all 19 investigated phyla. Source data

Consistent with the concept of niche conservatism, we postulated that related OTUs would tend to inhabit similar niches. To test whether related OTUs are indeed found in similar habitats, we used MicrobeAtlas environmental annotations to select five diverse main environments covering many samples: soil (*n* = 204,329), animal (*n* = 594,104), plant (*n* = 130,212), marine (*n* = 133,837) and freshwater (*n* = 38,414). We implemented prevalence-based majority voting to assign each OTU to one of these five primary environments. Our analysis revealed a consistent trend for related species to be found in the same main environment, partially driving the observed community conservatism trends (Fig. 4a). These findings suggest that broad-scale niche conservatism, the tendency of OTUs to remain in their primary environments, is also evident in microorganisms. While we used only broad, diverse habitat classifications, previous observations of niche conservatism at a smaller scale^25,59^ indicate that this concept could extend to more specific ecological niches.

We were furthermore curious whether community conservatism extends beyond these broad environmental preferences, that is, whether it still persists even when the primary environment is normalized for. In addition, we hypothesized that microorganisms also tend to keep similar ‘partners’, for example, in mutualistic relations or by preferring certain abiotic factors that extend beyond the traditional definitions of a niche. For instance, we expected that when mitigating the environmental effect, any remaining differences would mainly reflect ‘interaction conservatism’ (phylogenetic assortativity). To investigate whether the community conservatism signal remains when accounting for broad environmental preferences and to quantify differences across environments, we repeated our workflow with OTU pairs that were predominately found in the same main environment or to pairs belonging to different environments (Fig. 4b).

This analysis revealed that community conservatism is consistently observable even within different environments, interestingly to varying degrees. Conversely, as expected, OTU pairs annotated to different main environments had the lowest overall community similarities—but still with a clearly visible community conservatism signal. Most environments showed similar BCS ranges of their OTU pairs, with the notable exception of soils, which showed almost twice the community similarity of other environments.

**Extended Data Fig. 7:**
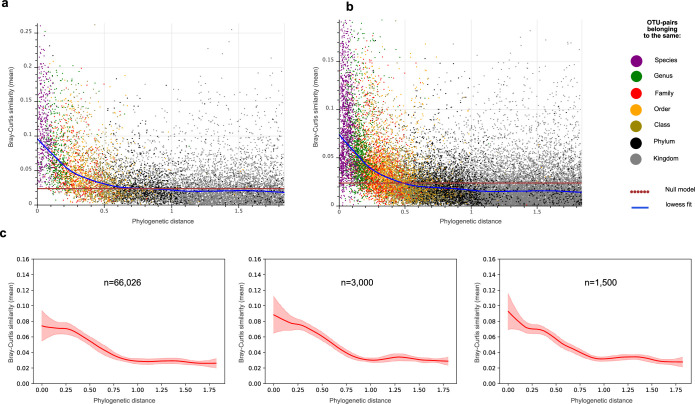
Community conservatism is robust to potential confounding factors. **a**. The same OUT-pairs as in Plot 3a are shown based on a rarefied subset of samples, downsampled to 10,000 reads. The original null model is shown. **b**. In this plot, the alpha diversity (richness) of all samples is reduced to the 50 most abundant OTUs. **c**. Trendlines of animal OTUs are shown with the original number of OTUs (n = 66,026), and two reduced sets (n = 3,000 and n = 1,500). The solid line shows the mean of 30 bootstrapped lowess fits. Shaded areas denote 1.96 x standard deviation (approximate 95% confidence interval). Source data

While soil microbial communities can vary substantially even at centimetre scales and show the highest OTU richness, they are globally more similar than often assumed and are usually dominated by relatively few OTUs, which would lead to high community similarity values^60,61^. Interestingly, the community conservatism of OTUs mainly annotated to plants already plateaus at approximately genus-level phylogenetic similarity. This could potentially be rationalized by OTUs having ‘locked in’ preferences for certain plant types or for different plant areas (root or shoot) already at a broader phylogenetic level. The environments differ in their alpha diversity and sequencing depth, which may impact our results by shifting beta diversity values systematically. In our dataset, we found that altering sequencing depth did not influence the overall beta diversity values (Extended Data Fig. 7a). By contrast, artificially reduced richness resulted in an overall lower BCS—probably owing to the absence of shared, rarer taxa found in many samples (Extended Data Fig. 7b). The general trend, however, remained stable in all tested scenarios. It is important to note that only 2,781 OTUs are predominately annotated to plants, which is fourfold less than in any other environment (next lowest: freshwater, 11,671 OTUs). To verify whether the smaller number of OTUs in plants could have caused the observed plateau, we reduced the animal environment to a similar number of OTUs (1,500 and 3,000). The trend line remained almost identical, indicating that the plateau of plant OTUs is probably due to true biological distributions and not driven by the lower number of plant OTUs (Extended Data Fig. 7c).

### Phylum-specific characteristics of community conservatism

**Extended Data Fig. 8:**
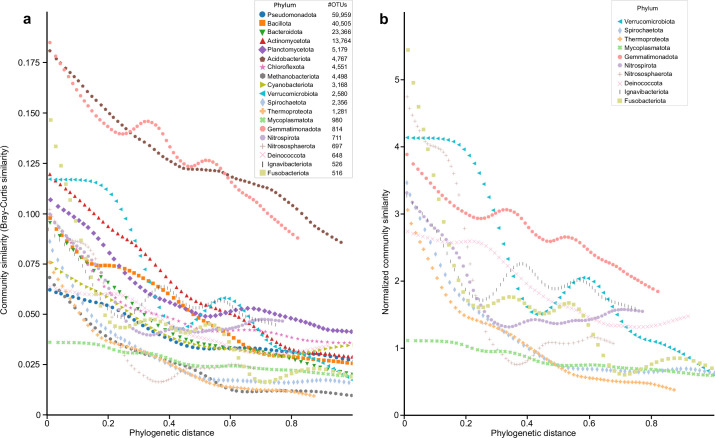
Microbial phyla differ in shape and strength of community conservatism. **a**. Lowess trendlines (non-normalized) of all phyla with >500 OTUs are shown here. Each lowess fit stems from 10,000 OTU-pairs. **b**. Normalized lowess trendlines of all the additional phyla with >500 and <3000 OTUs are shown here. Each lowess fit is calculated from 10,000 OTU-pairs each. Each phylum is separately normalized according to Supplementary Table 1 (See Methods). This panel shows an increased level of noise in the form of bumps when comparing it to Fig. 4c (normalized phyla trendlines with >3000 OTUs).

**Extended Data Fig. 9:**
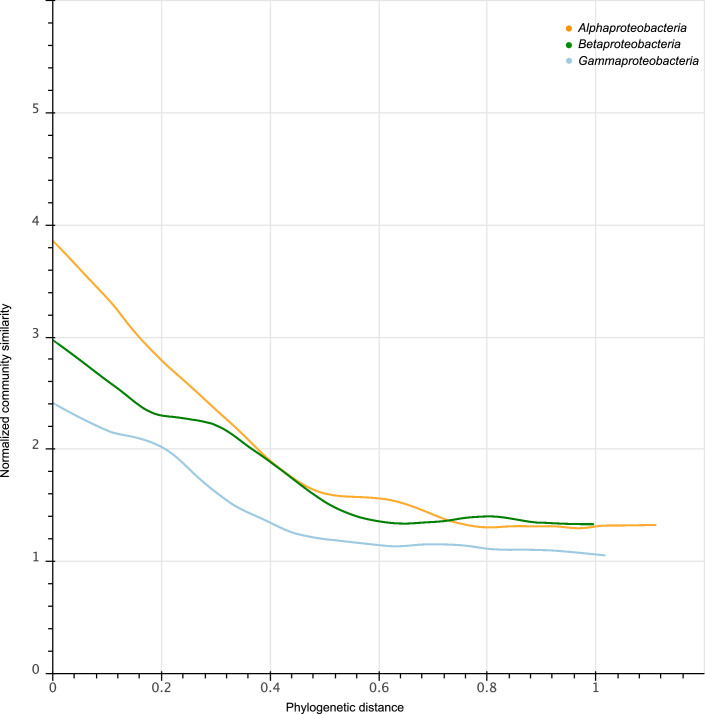
Normalized trendlines reveal differences among three Pseudomonadota classes. Lowess trendlines of the three classes Alphaproteobacteria, Betaproteobacteria and Gammaproteobacteria are shown. Each lowess fit is calculated from 10,000 OTU-pairs. Each class is separately normalized according to Supplementary Table 1 (See Methods).

We showed that community conservatism is present in microorganisms on a global scale, irrespective of their main environment, and extending as far back as the phylum level. The next question we wanted to address is whether we can infer characteristics of the ecology, speciation and community assembly processes across the different phyla. For this, we next repeated the previous analysis separately for each phylum represented by a minimum of 500 OTUs in the MicrobeAtlas database, while also calculating an individual phylogenetic tree for each phylum. In total, these were 3 archaeal and 16 bacterial phyla (Extended Data Fig. 8a). As we showed previously, the environment in which the phyla are mainly found strongly influences community conservatism. This is, for instance, visible in Acidobacteriota and Gemmatimonadota. Both phyla show a high average community similarity as they are predominately found in soil. To mitigate that environmental effect, we calculated phylum-specific null models, considering the expected community similarity values by accounting for the main environments of the compared OTUs (Supplementary Table 1). By normalizing our community similarity metrics against these phylum-specific baselines (Methods), we obtained normalized community conservatism curves. Intriguingly, these curves trend differently across phyla, with those containing less than 3,000 OTUs showing increased noise (Extended Data Fig. 8b). Yet, most phyla show a clear decrease in community conservation when assessing increasing taxonomic distances from the species level to the phylum level. We see a steep descent in some phyla (for example, Methanoproteota, Chloroflexota), while in others, the decrease is more gradual (Fig. 4c). We hypothesized that quantifying the steepness of the trend line, as well as differences to the baseline, would help us characterize ecological characteristics of each phylum: a steep curve would indicate recent ecological shifts: very closely related OTUs still share similar communities, but slightly more distant relatives already occur in different communities, suggesting that niche specialization arose between these points. As not all OTUs were taxonomically annotated to the species level, we instead used the density gradients obtained in Fig. 3c and binned the OTUs accordingly to approximate the taxonomic levels. In all investigated phyla, we observed highly significant (two-sided *P*_Mann–Whitney *U*_ < 0.05, Supplementary Table 2 and Fig. 4d) decreases in community conservatism when comparing the species level to the phylum level. We furthermore quantified the decrease of community conservatism from the species level to the genus level (Fig. 4d), reasoning that ongoing changes in community preferences should be reflected by differences in the species and genus levels. We found 15 phyla that were still significantly different in their community conservation when comparing the species level with the genus level (two-sided *P*_Mann–Whitney *U*_ < 0.05, Supplementary Table 2). For instance, Thermoproteota have large increases at both levels (species/genus ratio: 1.6; species/phylum ratio: 3.9), showing a strong and ongoing tendency to change communities and to specialize into different niches. All investigated archaeal phyla show sharp increases (species/phylum ratios > 2.8), aligning with their tendency to be found in extreme environments and their adaptability to new environmental factors^62^. However, phyla such as Acidobacteriota, predominately found in soils^63^, show a comparatively shallow increase (species/genus ratio: 1.1; species/phylum ratio: 1.6). This indicates that most members of this phylum have long been restricted to their respective niches and do not usually adapt and evolve quickly into new habitats or roles. For very OTU-rich phyla, for instance Pseudomonadota (former Proteobacteria), it is also feasible to investigate lower phylogenetic levels separately, such as Alphaproteobacteria, Deltaproteobacteria and Gammaproteobacteria. While the overall pattern of community conservatism remains evident at these finer taxonomic scales, the classes vary in the strength of the signal (Extended Data Fig. 9).

Our analysis of community conservatism provides a quantifiable measure of ecological similarity among related OTUs, a concept central to methods such as UniFrac^16^. UniFrac compares communities by considering phylogenetic relationships through tree branch length calculations^16^ (that is, closely related species are assumed to be similar and thus to contribute less to diversity). However, UniFrac defines relatedness for all microorganisms equally, while our study reveals that different phyla show varying rates of community similarity with decreasing relatedness. We propose that the values presented in our analysis, or alternative metrics of ecological similarity within microbial phyla, could be used to develop a more ecologically resolved version of UniFrac in the future. This enhanced method would apply taxon-specific weights (depending on the ecological similarity) when aggregating tree branch lengths, potentially offering a more nuanced approach to community comparison. In practice, this would assign greater weights to closely related taxa that are ecologically divergent (indicating rapid niche shifts) while down-weighting distantly related taxa that nevertheless share similar communities.

### Specialists and generalists have distinguishable community conservatism trends

Conceivably, the observed differences between phyla in terms of community conservatism might hint at general differences in their degree of ecological specialization: when members of a phylum show little specialization (that is, they are generalists), we would expect their communities to be fairly diverse, with community–community distances averaging out at a certain level set by the overall diversity of the available data. Conversely, in phyla predominantly composed of specialists, closely related pairs would be hypothesized to share very similar communities, whereas the communities of pairs with larger phylogenetic distances are expected to be very dissimilar as they occur in very distinct niches.

**Fig. 5:**
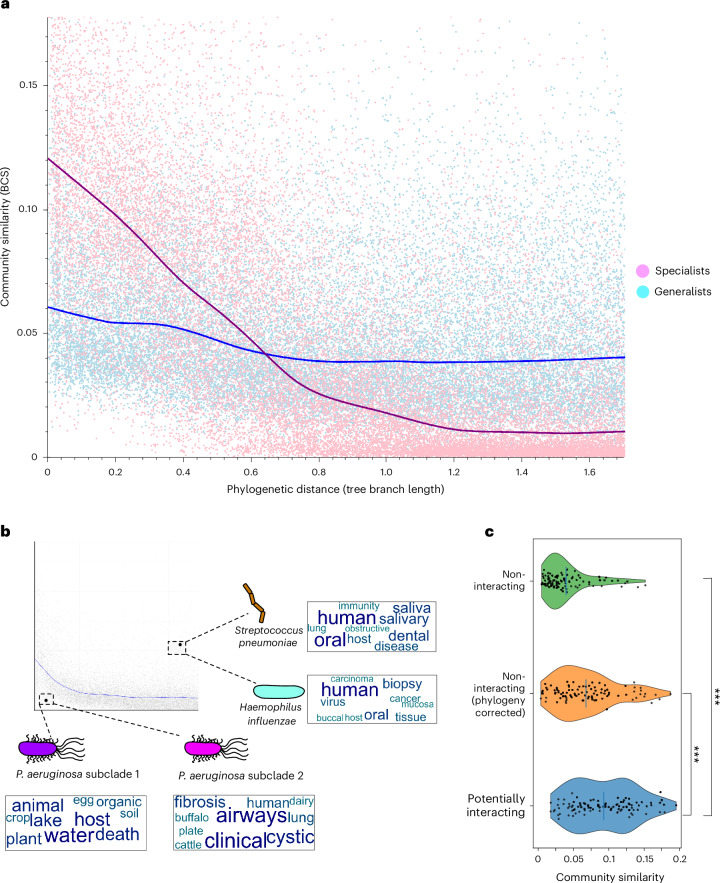
Community conservatism correlates with ecological properties. **a**, A total of 25,000 OTU pairs consisting of only generalists are compared with 25,000 specialist pairs. Lowess fits of both groups (pink and light blue trend lines) are plotted on top. **b**, Two outlier OTU pairs are highlighted here: two closely related *P. aeruginosa* subclades and *H. influenzae* and *S. pneumoniae*. The OTUs are annotated with the most common keywords obtained from the metadata of their global distributions. We supply all further outlier pairs in Supplementary Tables 4 and 5. **c**, Violin plots depicting the community similarities of OTU pairs that are predicted to interact based on FlashWeave (*n* = 100, blue violin plot), the same number of randomly selected pairs (green) and random pairs corrected for phylogenetic relatedness bias (orange). Vertical lines denote the mean in each violin plot. ***Potentially interacting pairs compared with non-interacting: two-sided *P*_Mann–Whitney *U*_ = 1.1 × 10^−17^; comparing with the phylogeny corrected set: two-sided *P*_Mann–Whitney *U*_ = 4.7 × 10^−5^. Source data

To check for this, we first devised a habitat generalism score for each OTU, based on their normalized abundances across different environments (Methods). We then selected the top 10% OTUs (‘generalists’) and the bottom 10% OTUs (‘specialists’) and calculated the community conservatism of both groups. Strikingly, the results reveal a clear separation: generalists show small, steady increases of community conservatism, with a relatively high baseline even in non-related pairs, whereas specialists show a much steeper trend line, with non-related pairs found in very different communities, whereas closely related pairs appear in very similar communities (Fig. 5a).

Applying this observation to individual phyla (Extended Data Fig. 8 and Fig. 4c), we are tempted to speculate that phyla with shallow increases in community conservatism, such as Pseudomonadota and Mycoplasmatota, could be more generalist in nature. In contrast, phyla with specialist-like trend lines, such as Fusobacteriota and several archaeal phyla, might indeed have specialist lifestyles. Similar to ref. ^22^, we hence leverage community composition data to infer generalist and specialist phyla—but here with a pairwise OTU comparison approach. For this, we compare pairs of OTUs to assess whether phyla with distinct generalism and specialism scores show different trends of community conservatism. Indeed, per-phylum aggregated habitat generalism scores are significantly correlated with curve steepness (*r* = 0.46, *P*_P__earson_ = 0.045; Supplementary Table 3). These scores show similar correlative trends with the phylum-specific social niche breadth scores of ref. ^22^, albeit not quite significant (*r* = 0.41, *P*_P__earson_ = 0.079; Supplementary Table 3). These results give support to both the social niche breadth metric and our use of curve steepness to independently infer generalist and specialist phyla based on community composition.

### Outliers can be ecologically informative

Most of the sufficiently sampled OTUs conform to the trends above—but it may also be interesting to look at outliers: pairs that are closely related but dissimilar in their communities are hinting at relatively recent evolutionary pressures to change niches. Conversely, distantly related OTU pairs that are similar in their communities might depend on each other or have a shared niche requirement independent of phylogeny. We provide a list of both types of outliers (Supplementary Tables 4 and 5) and highlight two examples in detail below (Fig. 5b).

On the bottom-left corner of the overall distribution plot are two *Pseudomonas aeruginosa* subclades that are closely related. Yet, against global conservatism trends, they occupy different communities, hence hinting at a strong ecotype difference between both subclades. To better understand their respective niches, we investigated all samples in which the OTUs are detected through metadata keyword summaries. This analysis indicates that *P. aeruginosa* clade 1 is adapted to the human host and enriched in samples of patients with cystic fibrosis, while *P*. *aeruginosa* clade 2 is a generalist found in many non-human environments. This overlaps with existing research showing that *P. aeruginosa* can be found in both niches^64–66^. However, the OTU pair of *Haemophilus influenzae* and *Streptococcus pneumoniae* are only distantly related, belonging to different phyla. Nevertheless, they share many community members and are both abundant in the human oral cavity and lungs (Fig. 5b), where they occasionally even form biofilms together^67^.

These and other examples led us to the hypothesis that community similarity is informative when identifying ecologically interacting OTU pairs: OTUs with more similar background communities should be more likely to interact. To investigate this, we analysed all investigated OTU pairs with FlashWeave, a software package that statistically predicts potential ecological interactions between OTUs^21^. And indeed, OTU pairs predicted to interact this way show much higher community similarity (*P*_Mann–Whitney *U*_ = 1.1 × 10^−17^, Cohen’s *d* = 1.37), also when correcting for phylogenetic relatedness (*P*_Mann–Whitney *U*_ = 4.7 × 10^−5^, Cohen’s *d* = 0.56; Fig. 5c). Together, these observations and the underlying data could prove useful to improve the inference of interacting or niche-defining OTU pairs.

## Outlook and conclusion

### Reintegrating ecological information into OTU delimitation in the future

How to best cluster bacterial and archaeal lineages into meaningful units that resemble a species is still under debate. Some argue for a strict operational approach using phylogenetic marker genes, usually by implementing a chosen species-level threshold (for example, 97% for 16S rRNA, 96.5% for average nucleotide identity (ANI) of the whole genome)^68,69^. Others argue that this procedure is too simplistic and that phenotypic and ecological information should be considered as well^70^. In any case, most agree that delimiting species-level clusters using the same specific thresholds is pragmatic and operational, but not always ideal^71^. Using the full genome as in the Genome Taxonomy Database is probably the best way of delineating microorganisms, but many microorganisms still do not have an associated genome: in MicrobeAtlas, only 11.3% of the 111,870 OTUs (97% level) are covered by genomes in ProGenomes3 and BacDive^35^. In addition, 16S-based amplicon sequencing is still the predominant method of analysing microbial datasets (almost tenfold increase over other technologies^35^). Hence, it will also be crucial to improve the delimitation of taxonomic groups for which only 16S sequences are available: while in some cases bacterial strains that belong to the same OTUs may differ strongly in their environmental role, others might be traditionally assigned to two different OTUs, while performing the same principal role in the ecosystem.

**Fig. 6:**
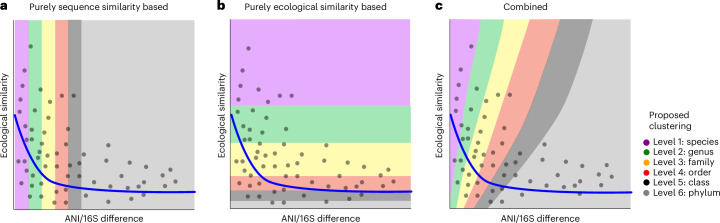
Reintegrating ecological information into OTU delimitation. **a**, Each grey dot denotes a hypothetical pair of lineages (strains). The classical assignment of lineages into OTUs takes only sequence similarity into account (usually, 16S rRNA similarity or ANI). **b**, A hypothetical classification of lineages based only on ecological niche similarity. **c**, We propose a combination of both as a more realistic system: reintegrating ecological information (such as community conservatism) into OTU clustering, considering both sequence similarity and ecological information when forming ecologically informed OTUs (eOTUs) using a multiphased clustering approach.

**Extended Data Fig. 10:**
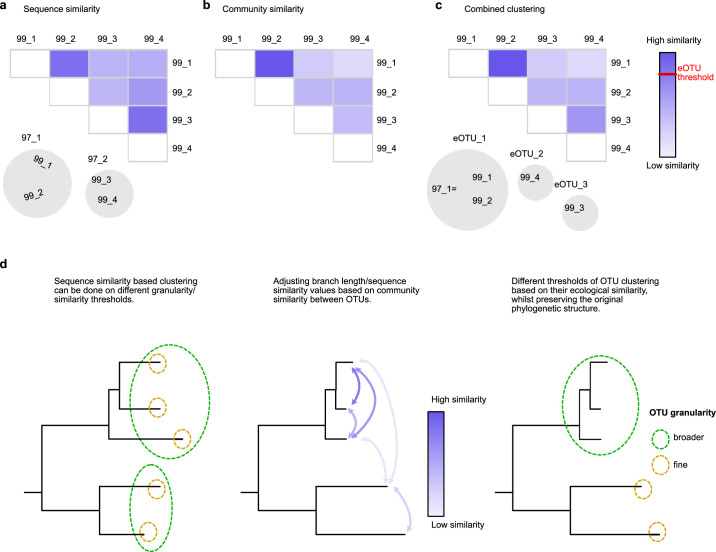
Conceptual framework for potential eOTU clustering. **a**. In this conceptual example, four 99% OTUs are closely related. Pair-wise sequence similarity values are shown in the illustrated table. When using a 97% clustering threshold in OTUs, 99_1 and 99_2 would cluster together into one 97% OTU; and 99_3 and 99_4 would form a second such OTU. **b**. Pair-wise Bray-Curtis similarities (BCS) are shown in the table. When investigating ecological information, it becomes apparent that 99_1 and 99_2 are very similar in their niches, whereas other pairwise comparisons point to diverse habitats/ecological preferences. **c**. We propose to join both metrics to inform the potential definition of ecological OTUs: eOTUS. In this hypothetical example, a to-be-determined eOTU threshold delimits the four 99% OTUs into three ecologically consistent eOTUs. More specifically, considering the environmental information would result in an alternative clustering that groups the environmentally similar OTUs 99_1 and 99_2 into one eOTU. On the other hand, 99_3 and 99_4 appear to occupy different niches and would thus be considered as their own respective eOTUs. **d**. A different schematic representation of this approach with five fine scale (for example 99%) OTUs, emphasizing the constraints by existing evolutionary and phylogenetic relationships. The phylogenetic branching based on sequence similarity values can define OTUs of various granularity. Branch-lengths can be adjusted by their respective community similarity values, resulting in a combined strategy where clustering thresholds are more ecologically meaningful and enable a multi-phased OTU-clustering into eOTUs of different granularities.

Previous research has argued that a distribution-based approach could be used to improve OTU delimitation^72,73^. Here we propose to build upon these ideas and, instead of solely relying on marker similarity (Fig. 6a), to reintegrate ecological information into OTU delimitation. More specifically, we suggest achieving this by incorporating community conservatism information (Fig. 6b), resulting in a combined clustering strategy (Fig. 6c). Operationally, we envision a two-step OTU clustering approach (Extended Data Fig. 10): first, a purely sequence-based OTU clustering on the finest level (99%) would serve as an initial cursory analysis point that also provides a phylogenetic scaffold. In a second step, pairwise ecological similarities could be incorporated as an additional weighting to define ‘ecologically informed OTUs’ (eOTUs). Importantly, this approach is not intended to merge unrelated lineages, but rather to select the appropriate granularity to split overly inclusive sequence-based clades (for example, 97% OTUs) that may in fact contain ecologically distinct groups. This potential multiphased approach would be constrained by the original evolutionary phylogeny of a larger taxonomic group and then use the pairwise ecological similarities (altering only the branch lengths) to define eOTUs. For instance, if two 99% OTUs were found in different environments and thus dissimilar communities, we would expect them to occupy diverse niches and fulfil different roles, hence assigning them both to individual eOTUs. However, if a group of 99% OTUs appear very similar in their environments and co-occupants, we hypothesize that they would also perform a similar function in nature—thus retaining their broader 97% clustering as one eOTU (Extended Data Fig. 10d). This combined approach could yield a more natural OTU clustering, ideally combining advantages of phenotypically and ecologically informed taxonomy and purely sequence similarity-based OTU clustering. The choice of how highly to weigh sequence similarity versus community similarity will be subject to empirical and theoretical considerations. Similarly, while we here used pragmatic, data-driven measures for sequence identity and community similarity, the choice of metrics is flexible. Future work on this will require fine-tuning, benchmarking and comparisons to genome phylogenies that are outside the scope of this study. For now, we wish to highlight that community preferences and their conservation trends are easily assessed from cross-sectional data (in contrast to other relevant phenotypes) and show promise for more ecologically meaningful OTU delimitation.

## Conclusion

We found that community conservatism is present in all investigated phyla and environments, on a global scale. We postulate that this community conservatism signal could be useful to infer how quickly members of a given microbial lineage usually adapt to new environmental conditions (or communities). Potential applications include microbiome engineering, in which such inferences could improve predictions of species addition or removal effects in a given community, based on their niches^74^.

Our analysis is mostly based on 16S rRNA, which comes with some implications. Barely measurable divergences of 16S sequences can often reflect a substantial evolutionary divergence (1% divergence corresponds to millions of years)^75^, differing between phylogenetic lineages. One microbial genome may also contain more than one divergent copy of the 16S rRNA gene^76,77^. In addition, horizontal gene transfer occurs frequently between closely related microorganisms^78^ and, occasionally, horizontal gene transfer can occur even in 16S rRNA genes^79^. While those aspects have the potential to affect our analysis, they should (if anything) rather weaken the observed signal: if, for instance, horizontal gene transfer of 16S occurs, we might erroneously compare a ‘distantly related’ OTU pair as very closely related. Despite this, we consistently observe community conservatism across different phyla, timescales and environments.

Niche conservatism and phylogenetic signal are well-established concepts in the study of animals and plants, but their assessment in microorganisms has been limited by the challenges in ascertaining phenotypes and niches in free-living organisms. The concept of community conservatism offers an alternative approach to investigating these patterns in microbial communities, as well as the prospect of reintegrating ecological information into OTU delimitation.

## Methods

### MicrobeAtlas data retrieval

We used samples processed within the MicrobeAtlas project^35^. Briefly, we searched the NCBI Sequence Read Archive^80^ for samples and studies containing any of the keywords ‘metagenomic’, ‘microb*’, ‘bacteria’ or ‘archaea’ in their metadata and downloaded the corresponding raw sequence data. Raw data were quality filtered by discarding reads with low-quality bases. We additionally excluded samples containing less than 1,000 reads and/or less than 20 OTUs defined at 97% 16S rRNA gene identity, and further retained only samples with at least 90% estimated community coverage. The total filtered set amounted to 1,153,349 samples. Community coverage of in-reference OTUs was extrapolated using formula 4a in ref. ^81^ (based on an improved version of the Good–Turing frequency estimator). To assign OTU labels, quality-filtered data were mapped using MAPseq v.2.2.1 at a ≥0.5 confidence level^36^. Furthermore, we removed all eukaryotic reads to solely focus on the prokaryotic diversity.

The MicrobeAtlas project contains multiple hierarchically clustered OTUs at different sequence identity thresholds (90%, 96%, 97%, 98% and 99%) as described in ref. ^36^, resulting in hierarchical OTU definitions (parents and children). Clustering was performed using HPC-CLUST^39^.

NCBI Sequence Read Archive sample metadata were parsed to classify every sample into five general environments: animal, marine, freshwater, plant and soil. If a sample was assigned to more than one main environment (for example, ‘animal|soil’), it was counted for both environments; if it had no assignment, it was not counted for the environmental calculations. The environmental keywords ‘sea’, ‘ocean’ and ‘marine’ were combined into marine, and ‘lake’, ‘river’ and ‘freshwater’ into freshwater. Each OTU was then also assigned a main environment, based on a majority vote of sample prevalence. Environmental assignments and keywords of all samples can be found in the file ‘samples.env.info’ obtained from https://microbeatlas.org/index.html?action=download, on 10 March 2023. The identifiers of all specifically named OTUs used in the figures (Figs. 2 and 5b) are provided in Supplementary Table 6.

### Selection of OTU pairs, exclusion criteria

We used stringent criteria in selecting the OTUs (at a 99% identity threshold) and samples that we analysed. We compare only samples that do not belong to the same project ID. Each OTU is allowed only in a maximum of 9 comparisons (increased to 30 in phyla and environments <3,000 OTUs) to avoid overrepresentation of certain taxonomic groups.

For the general trend, we compared 25,000 pairs (formed by ~14,000 OTUs); we used an equal number of pairs for the environment-specific pairs. For the phylum-specific points, we used 10,000 pairs each. To obtain a uniform distribution of distances, we created 50 bins of phylogenetic distances and filled each bin with randomly drawn pairs within that reach. We removed the furthest 3% of distances (that is, the most distantly related pairs), as they may contain some misclassified OTUs, or cases in which the bin would otherwise be impossible to fill (Supplementary Fig. 1). The taxonomy of the OTUs was assigned according to the NCBI assignments of the representative 16S rRNA sequences.

### Phylogenetic tree generation

All full-length 16S rRNA gene reference sequences from MAPref v.2.2.1 were aligned with Infernal^36^. A large, phylogenetic tree of all OTUs was generated from the alignment using fastTree 2.1.10 with the ‘-nt -gtr -gamma’ parameters^82^, and multifurcations were removed subsequently using the resolve_polytomy (recursive=true) function in ete3 version 3.1.2 (ref. ^83^). To increase precision and avoid rare misplacements of some lineages in the universal phylogenetic tree, phylum-specific trees for each phylum with >500 OTUs were generated with the same evolutionary model to ensure comparability. Tree distances were extracted using the distance function of ete3.

### Fraction of shared samples and sequence similarity

For all OTU pairs that were compared in their tree distances and community similarities, we additionally calculated the sequence similarities of the full-length representative 16S rRNA sequences with a custom script. We furthermore calculated the fraction of shared samples based on the overlap in prevalence within MicrobeAtlas.

### Calculation of community similarities

The BCS (also called the quantitative Sørensen–Dice index) was calculated using the formula 1 − Bray–Curtis dissimilarity. HPC-CLUST v1.1.0 (ref. ^39^) was used for the calculations with the following parameters: ‘-t samples -nthreads 30 -dfunc braycurtis_skipproj -makecluststats -projf’. We repeated the analysis with the ‘-minlogfrac’ parameter to calculate log-transformed BCS. For each OTU pair, we compared all samples that do not belong to the same research project (that is, do not share the same ‘project ID’ at the Sequence Read Archive) in which they are detected in a pairwise manner (for example, if OTU 99_1 is found in samples A and B, and OTU 99_2 is detected in samples A, C and D, we would compare the community similarities of A–C, A–D, B–C and B–D; A–A would not be compared). We record multiple quantiles but use the mean in all plots unless specified otherwise. We used 90% OTUs for the computation of community similarity values. The output was further processed with pandas v1.0.3 (ref. ^84^) and plotted with bokeh version 2.2.3. We used a locally weighted scatter plot smoothing (lowess, statsmodel.api.nonparametric.lowess, frac = 1/5) as well as an exponential decay function (scipy.optimize.curve.fit) to fit the data. For quality control, we repeated the analysis twice: while rarefying all samples to 10,000 reads (discarding samples with a lower number) and furthermore by restricting the richness to the 50 most abundant OTUs per sample. Final plots were adjusted using Affinity Designer. All custom code is available via GitHub at https://github.com/lukasmalfi/community_conservatism.

### Null model generation

To create a general null model, we compared the communities of 50,000 randomly chosen sample pairs with the same parameters as described above. We verified this baseline by randomly picking the average number of samples (*n* = 2,150) of an OTU pair 1,000 times and averaging the resulting baselines. We furthermore created individual baselines for all environmental combinations (that is, comparing only soil–soil samples, animal–animal, animal–soil and so on). We then used these values (Supplementary Table 1) to generate phylum-specific baselines. There, we estimate the primary environment of each OTU in the pair and record their combinations (for example, a soil-associated OTU paired with an animal-associated OTU would be classified as ‘soil–animal’). We then computed the ratios of their environment combinations of the OTU pairs (for example, 10,000 pairs: animal–animal: 1,000 pairs: 0.1, animal–soil: 0.85, animal–aquatic: 0.05) and calculated the respective null model (0.1 × animal–animal baseline + 0.85 × animal–soil baseline + 0.05 × animal–aquatic baseline). For the community similarity values of different phyla, we normalized those by dividing the mean community similarity values by the calculated phylum-specific null model.

### Phylum-specific ratios

As many of the OTUs are not taxonomically annotated to species or genus level, we estimated the approximate range of species and genus OTU pairs from the general trend. We used the middle 60% of rank-specific distributions (that is, excluding the top and bottom 20%, respectively) to obtain ‘species-level’, ‘genus-level’ and ‘phylum-level’ bins based on the phylogenetic distance. We then calculated the average community similarities of those three bins for each phylum. As a next step, we divided each species-level bin by the other two to create the ratios used to estimate the increase in community similarity from the genus level to the species level, and from the phylum baseline to the species level.

### Outlier OTU pairs

We classified OTU pairs as outliers on both extrema: (1) pairs that are very closely related (tree branch length < 0.2), yet very different in their communities (mean BCS < 0.04), and (2) pairs that are quite distantly related (tree branch length > 0.8), yet their communities are similar (mean BCS > 0.08). In addition, we considered only outliers for which at least 10,000 sample comparisons had been calculated. We provide a list of all outliers that fall into these bounds in Supplementary Tables 4 and 5.

### Generalist and specialist analysis

We calculated a generalism metric related to Levins’ breadth, an ‘environmental flexibility’ index, for each OTU based on its abundance distribution across animal, aquatic, soil and plant environments^35^. In brief, for each OTU, average relative abundances were computed for each environment and normalized to sum to 1. Then, the Shannon entropy over these proportions was computed, yielding a generalism score that increases for more uniform abundances across these environments (indicating greater generalism) and decreases for OTUs with uneven abundances (suggesting more specialized adaptations). In Fig. 5a we plotted 25,000 ‘specialist’ OTU pairs (lowest environmental flexibility score) and 25,000 ‘generalist’ OTU pairs (highest environmental flexibility score). The individual generalism scores of all 99% OTUs with taxonomic annotations were aggregated to obtain phylum-level generalism scores. These were correlated with the increase of community conservatism from species to genus level (ratios) with a Spearman correlation using the stats.spearmanr function of the scipy package v1.4.1.

### Connection to ProGenomes3 and gene number analysis

To connect our OTUs to genomes, we mapped OTUs defined at 99% to the ProGenomes3 database^85^, containing almost one million bacterial genomes. For each genome, genes were called and counted by running Prodigal^86^ (v2.6.3) with the following parameters: translation table 11 (-g 11), closed ends (-c), treat runs of N as masked sequence (-m) and single procedure (-p single). Out of the genomes, 753,909 representative 16S rRNA sequences were extracted using barrnap and mapped with MAPseq v2.2.1 to 99% MicrobeAtlas OTUs to obtain the number of genes per OTU. We then repeated our main analysis workflow to estimate relatedness and community similarities. The trend of community conservatism remains stable when using only OTUs with a genome link (Supplementary Fig. 3a). When analysing the number of genes per genome, we found that more closely related OTU pairs also have a more similar number of genes (Supplementary Fig. 3b). All genome mappings are available for future studies (Supplementary Table 7).

### Hawaii Ocean Time series

We selected all samples belonging to the HOT series project ‘SRP092796’. These samples were collected from HOT cruises from August 2010 through April 2016 at the North Pacific Subtropical Gyre at Station ALOHA. We selected all 99% OTUs with >10% prevalence and calculated relative abundances in each sample. We then calculated Pearson correlation coefficients of all pairwise abundance profiles (corrcoef function of numpy 1.18.1) and pairwise phylogenetic tree branch lengths as described earlier. In addition, we calculated the pairwise community similarity of 5,000 uniformly selected (phylogenetic distance) marine OTU pairs with minimum prevalence of 10% in the HOT series as described previously. We created hexagonal binned plots to visualize our results with matplotlib.

### Word clouds

For each OTU, keywords of all samples in which they were found were added to a list using custom code in Python 3.7.6. The list of obtained keywords was used to create a word cloud with WordCloud v1.5.0 using a custom colour map and the following parameters: stopwords = stopwords, prefer_horizontal = 1, min_font_size = 10, max_font_size = 150, relative_scaling = 0.4, width = 1000, collocations = False, height = 400, max_words = 15, random_state = 1, background_color = “white”.

### Interaction network analysis

We analysed the OTU pairs plotted in Fig. 3 by constructing a global network of predicted interactions. While FlashWeave uses co-occurrence, our main analysis pipeline excludes the co-occurrence signal, making the analysis thus orthogonal. We used the local-to-global learning approach^87^ using FlashWeave v.0.19.0 (ref. ^21^). This method generates a Bayesian network skeleton, representing potential ecological relationships between species while accounting for ecological or technical confounding factors.

FlashWeave’s algorithm operates in two main steps: first, it heuristically identifies likely confounding variables for each species pair based on univariate associations and previous algorithm iterations. Second, it tests whether the focal association persists when conditioned on these candidate confounders.

We configured FlashWeave with the following parameters: sensitive = false, heterogeneous = true and max_k = 3. With these settings, the software converts non-zero read counts to centred log-ratio-transformed values, addressing compositionality issues, and then discretizes these values. Conditional mutual information tests are subsequently performed on the discretized data.

We chose the 100 OTU pairs with the highest predicted interaction score to compare them against a random selection of 100 random OTU pairs from the same dataset. In addition, a second control group was chosen with a phylogenetic distribution matching the high-interaction pairs, to correct for phylogenetic relatedness. To this end, for each OTU pair selected, a random control within ±0.025 tree branch length was drawn.

### Statistics

The comparisons of the community similarity values of different taxonomic groups were performed using a two-sided Mann–Whitney *U* test in the scipy package v1.4.1 (‘stats.mannwhitneyu’)^88^. We calculated the differences between the interacting pairs and the control groups using a two-sided Mann–Whitney *U* test. Resulting *P* values were corrected for multiple testing using the Benjamini–Hochberg method. Effect size was calculated using Cohen’s *d*.

### Reporting summary

Further information on research design is available in the Nature Portfolio Reporting Summary linked to this article.

## Supplementary information

Supplementary Information Supplementary Figs. 1–3.

Reporting Summary

Supplementary Tables Supplementary Tables 1–7.

## Source data

Source Data Fig. 2 Source data for Fig. 2.

Source Data Fig. 3 Source data for Fig. 3.

Source Data Fig. 4 Source data for Fig. 4.

Source Data Fig. 5 Source data for Fig. 5.

Source Data Extended Data Fig. 1 Source data for Extended Data Fig. 1.

Source Data Extended Data Fig. 2 Source data for Extended Data Fig. 2.

Source Data Extended Data Fig. 4 Source data for Extended Data Fig. 4.

Source Data Extended Data Fig. 5 Source data for Extended Data Fig. 5.

Source Data Extended Data Fig. 6 Source data for Extended Data Fig. 6.

Source Data Extended Data Fig. 7 Source data for Extended Data Fig. 7.

## Data Availability

All data are available via Zenodo at 10.5281/zenodo.15689423 (ref. ^89^). For this study, we used an older version of MicrobeAtlas that can be downloaded via the same Zenodo link. Source data are provided with this paper.

## Code availability

All custom code used in the analysis can be obtained via GitHub at https://github.com/lukasmalfi/community_conservatism.
