# Supplementary material for: Community conservatism is widespread across microbial phyla and environments
Source: Nat Ecol Evol. 2026 Jan 16;10(2):232–45. doi: 10.1038/s41559-025-02957-4 (PMC12890590; doi:10.1038/s41559-025-02957-4)
Supplement: Supplementary file 1 — Supplementary Figs. 1–3. [file 41559_2025_2957_MOESM1_ESM.pdf]

nature ecology & evolution

Supplementary information https://doi.org/10.1038/s41559-025-02957-4

Community conservatism is widespread across microbial phyla and environments

In the format provided by the authors and unedited

Supplementary Figures

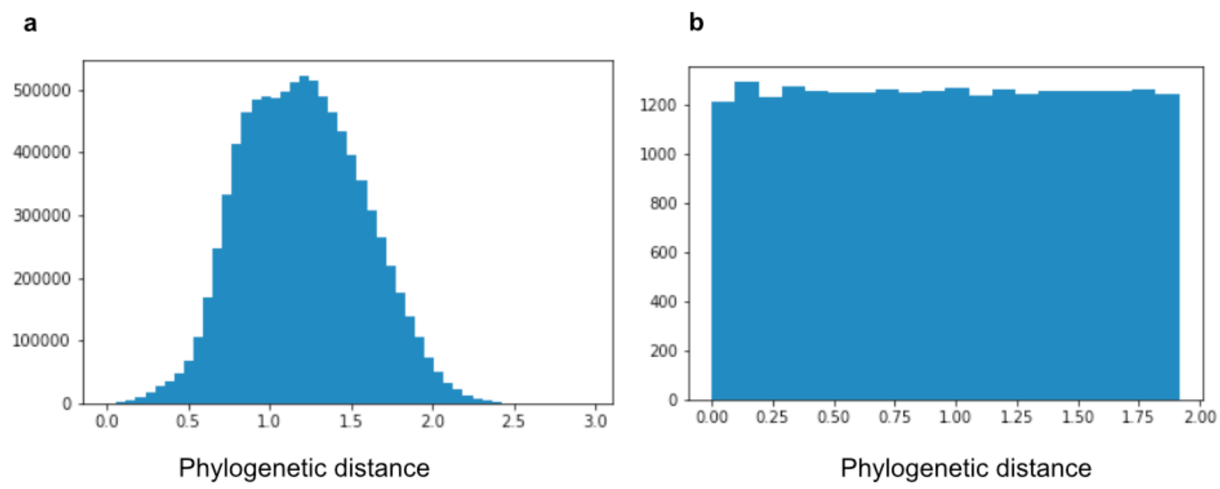

Supplementary Fig. 1: Obtaining a uniform distribution of phylogenetic distances

a. Downsampling the phylogenetic distances of OTU-pairs from the estimated original distribution to **b**. achieve a uniform distribution of phylogenetic distances over the whole range of relatedness.

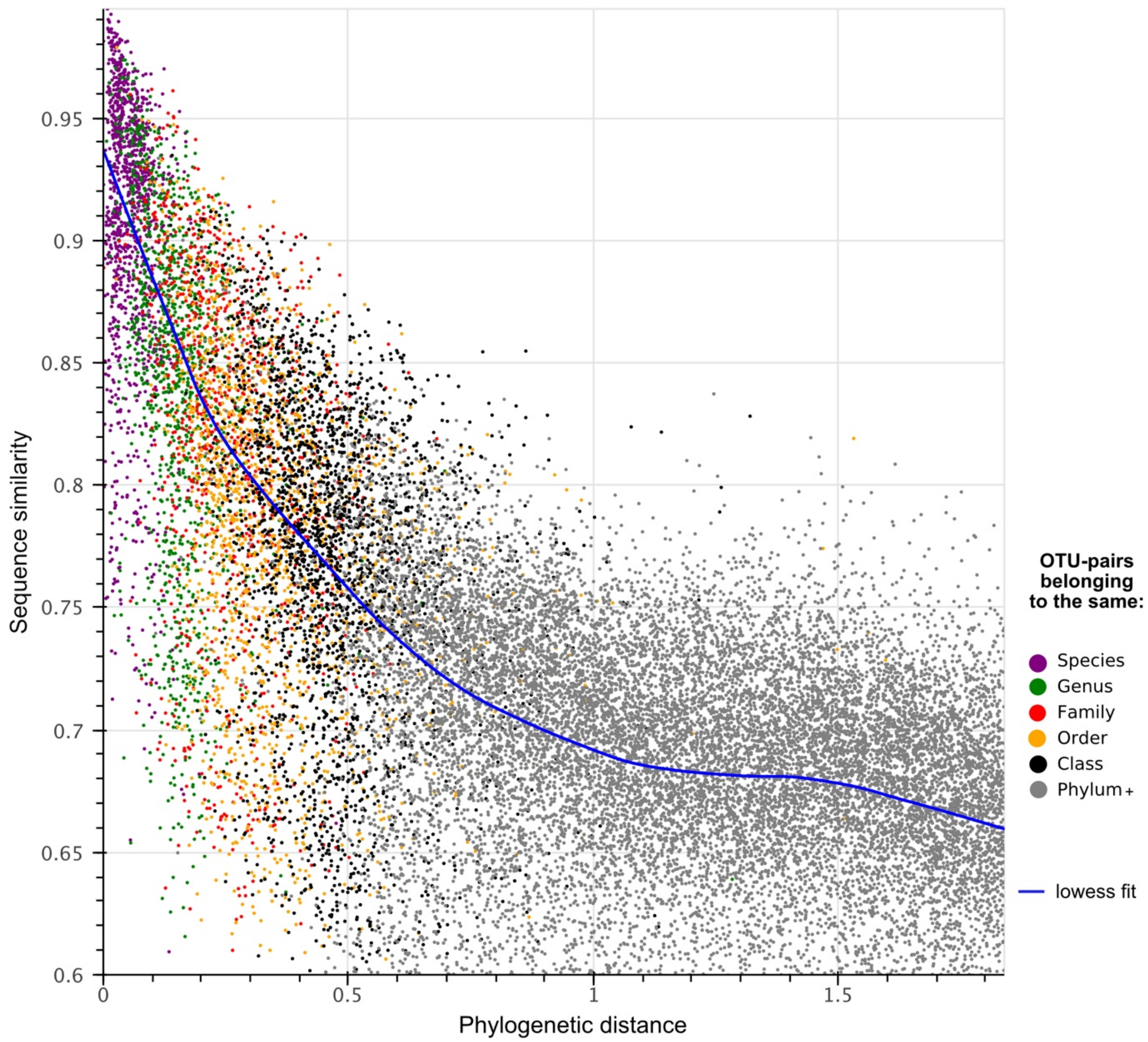

Supplementary Fig. 2: Sequence similarity correlates strongly with phylogenetic distance

The sequence similarity of the full-length 16S rRNA representative sequences of the OTU-pairs (y-axis) and the distance estimated by the tree branch length from the phylogenetic trees (x-axis) are plotted against one another. A lowess fit is applied to the data and shown with a blue line. Each dot corresponds to one OTU-pair colored according to their most specific shared taxonomic rank, with their relatedness shown on the x-axis and sequence similarity of their respective 16S sequences on the y-axis.

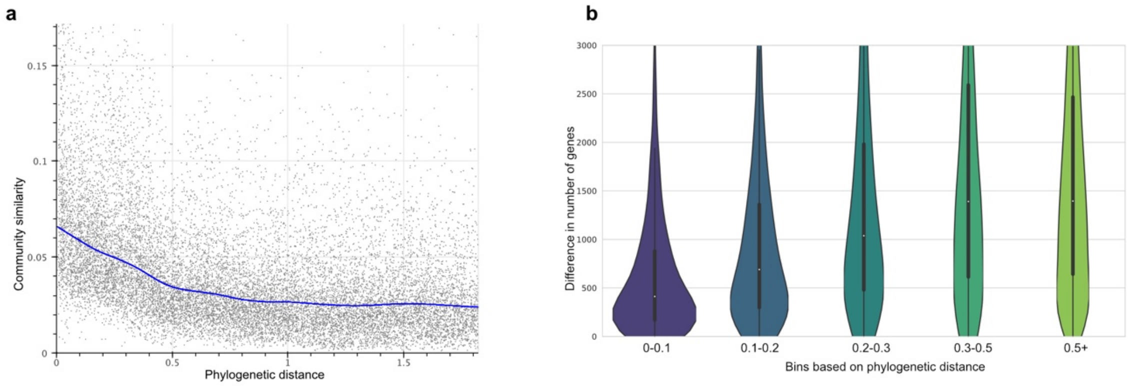

Supplementary Fig. 3: Closely related OTUs encode a similar numbers of genes

a. Community similarity falls as phylogenetic distance increases, visualized here through 16,000 OTU pairs with a mapped genome in the ProGenomes3 database; lowess fit is shown as blue line. **b**. The differences in the number of genes (y-axis) of OTU-from panel a are shown as violin plots, binned into 5 bins based on phylogenetic distances on the x-axis. The white dot denotes the mean, the black thick line interquartile range (25th to75th percentiles). All OTU-pairs with associated gene numbers are provided in Supplementary Table 6.
